# *Porphyromonas asaccharolytica* as a Rare Causative Agent for Lemierre's Syndrome

**DOI:** 10.1155/2018/3628395

**Published:** 2018-11-07

**Authors:** Mridul Gupta, Radhika Annam, Joseph Bahgat, Margaret Eng

**Affiliations:** ^1^Internal Medicine, Monmouth Medical Center, Long Branch, NJ, USA; ^2^St. George's University, West Indies, Grenada

## Abstract

Lemierre's syndrome is a rare disease associated with significant morbidity and mortality. It begins with an oropharyngeal infection, which spreads locally to involve the internal jugular vein causing thrombophlebitis, followed by distant spread and metastatic infections. Affected individuals are commonly young adults. Causative organisms are usually oropharyngeal flora, most commonly being the anaerobe *Fusobacterium necrophorum*. *Porphyromonas asaccharolytica* is a rare etiological agent with only three cases being reported in the literature. This case report describes a previously healthy 22-year-old man who initially presented with acute tonsillitis and was later found to have left internal jugular vein thrombophlebitis along with bilateral septic emboli to the lungs. The patient was treated with a five-week course of ampicillin-sulbactam and metronidazole. Subsequent imaging also showed progression of internal jugular vein thrombus, for which warfarin was given for three months for anticoagulation.

## 1. Introduction

Lemierre's syndrome is described as an acute oropharyngeal infection with secondary thrombophlebitis of the internal jugular vein leading to sepsis and metastatic infections [1]. It is also known by the terms such as “postanginal septicemia” and “necrobacillosis” [2]. This entity was much more common in preantibiotic era and was associated with a rapidly fatal disease course [3]. A number of cases of Lemierre's disease decreased drastically in the 1960s with widespread use of penicillins [4]. There has been a resurgence in the number of cases reported since the 1980s. Affected individuals are usually young, previously healthy adults. In most of the case series described for Lemierre's syndrome, males are more commonly affected as compared to females [3]. Causative organisms are usually oropharyngeal flora, most commonly being the anaerobe *Fusobacterium necrophorum* [2]. *Porphyromonas asaccharolytica* is a rare cause of Lemierre's syndrome with only a few cases being reported in the literature [5–7].

## 2. Case Presentation

A 22-year-old African American male initially presented to the emergency department with a three-day history of fever, chills, sore throat, and odynophagia. On examination, the patient was febrile with a temperature of 104°F and tachycardic. There was localized tenderness over the neck, and the patient had hyperemic and hypertrophic bilateral tonsils with whitish exudate. A CT scan of the neck with contrast showed acute tonsillitis with no peritonsillar abscess. Blood cultures and throat cultures were sent as part of a routine workup of a febrile patient. The patient was discharged from the emergency department on amoxicillin/clavulanate for acute tonsillitis. The patient was called back two days later, after his blood cultures showed growth for Gram-negative anaerobic bacteria. There was also growth of beta-hemolytic streptococcus on throat cultures. The patient had not taken any medications in these intervening two days.

**Figure 1:**
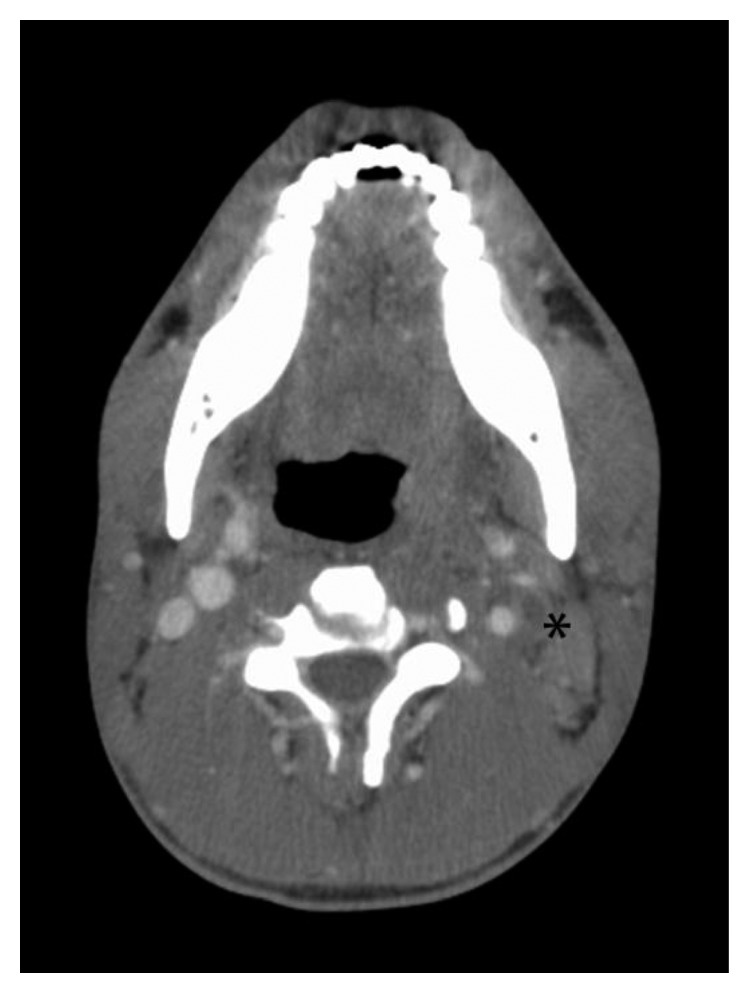
Contrast-enhanced CT scan of neck showing filling defect in left internal jugular vein (asterisk).

**Figure 2:**
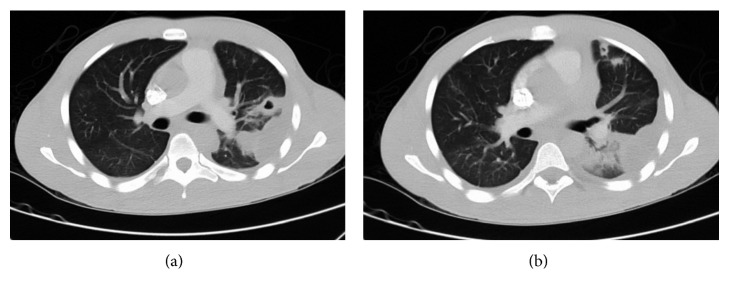
(a) Axial section (lung window) at the level of carina showing a peripheral patch of consolidation with central cavitation 2.8 × 1.6 cm in size, with a small pleural collection. (b) Axial section (lung window) showing 1.1 × 1.1 cm cavitary lesion, with 1.8 × 1.6 cm nodular density in lingula.

On readmission, a new set of blood cultures were drawn, and he was started on intravenous ampicillin-sulbactam. After five days of antibiotics, the patient had persistent fever, leukocytosis, throat pain, and dysphagia. On physical examination, the patient had increased localized tenderness over his neck, without any fluctuating mass. At this point, another CT scan of the neck with contrast was repeated to rule out any drainable collection. The repeat CT scan of the neck with contrast showed rim-enhancing left peritonsillar collections with adjacent thrombophlebitis (Figure 1). Also, an axial contrast-enhanced chest CT scan showed multiple new patchy cavitary nodules of the lung, suspicious for septic emboli (Figures 2(a) and 2(b)). Gram-negative anaerobic bacteria from the initial emergency department visit were later identified as *Porphyromonas asaccharolytica*. Repeat blood cultures did not show growth of any microorganism.

Intravenous ampicillin-sulbactam was continued, and metronidazole was added to the treatment regimen. In the setting of persistent pyrexia with evidence of left-sided peritonsillar collection, aspiration of the abscess was attempted with a 23-gauge needle but was unsuccessful. Due to persistent symptoms and fever spikes, despite appropriate antibiotic therapy, a repeat CT scan of the neck with contrast was pursued which showed progression of IJV clot. Thus, anticoagulation was initiated. The patient received anticoagulation with warfarin bridged with heparin for a total duration of 3 months. The patient was discharged home with a peripherally inserted central line to complete a 5-week course of antibiotic therapy.

**Figure 3:**
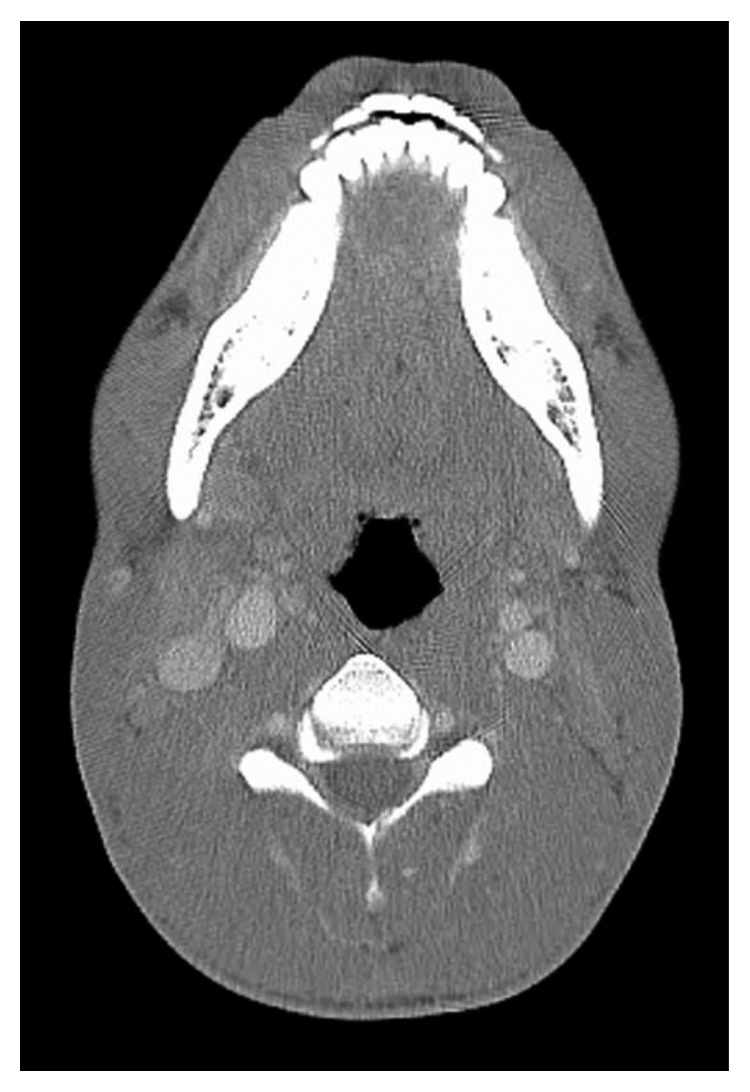
Resolution of left IJV filling defect after treatment.

**Figure 4:**
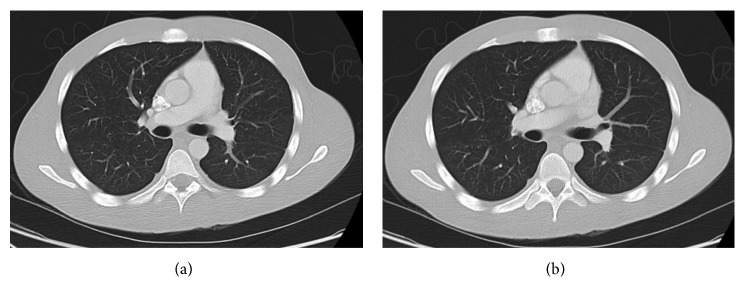
(a, b) Complete resolution of both parenchymal and pleural opacities on follow-up CT thorax following treatment.

The patient was initially lost to follow-up as he did not show up for follow-up appointments despite repeat reminders. However, he later presented to the hospital 8 months after the initial presentation with complaints of sore throat. CT imaging of the neck and chest was done at this point and was consistent with complete resolution of the internal jugular vein thrombus (Figure 3) as well as cavitary lung nodules (Figures 4(a) and 4(b)).

## 3. Discussion

Lemierre's syndrome was first reported by Courmont and Cade more than 100 years ago [8]. A more detailed description of this syndrome was given by Andre Lemierre in 1938 [9]. Lemierre described this new disease entity as “the appearance and repletion several days after the onset of a sore throat (and particularly of a tonsillar abscess) of several pyrexial attacks with an initial rigor, or still more certainly the occurrence of pulmonary infarcts and arthritic manifestations, constitute a syndrome so characteristic that mistake is almost impossible.”

There have been variations in the definition of this syndrome with some authors only considering disseminated *F. necrophorum* infections arising from the throat. Others have included oropharyngeal infections with metastatic spread to other organs, irrespective of foci of origin. In a recent prospective study from Denmark, the incidence of Lemierre's syndrome was found to be 3.6 cases per million inhabitants, contrary to previous estimates of only 1 case per million inhabitants based on retrospective studies [4, 10]. In the same study, the incidence in the age group of 15–24 was found to be 14.4 cases per million per year. It was rarely found in adults over 40 years of age. Slight seasonal accumulation was found with cases being more accumulated during late winter and early spring [10].

Presentation, epidemiology, and the natural history of this disease entity appear to have drastically changed over the years. Changes in patterns of antibiotic availability and usage along with the availability of better diagnostic testing have led to changes in trends of this disease. An increase in cases of this syndrome after 1980s has been attributed to a decreased trend of prescribing empiric antibiotics for sore throat, as well as the use of macrolide antibiotics which have no susceptibility to oral anaerobes. Nonsteroidal anti-inflammatory drug (NSAID) use and increasing antibiotic resistance especially to *β* lactams, Epstein–Barr virus (EBV), and other viral infections have all been described and proposed as possible reasons for increased number of cases [11–13]. On the other hand, there have been a number of studies disputing this fact, considering that the “reemergence” is just from improved diagnostic testing and blood cultures.

The most common microorganism associated with Lemierre's syndrome is *Fusobacterium necrophorum* [2]. Analysis of published cases from 1970 to 2007 done by Riordan showed that 86% of the total cases of Lemierre's syndrome had shown growth of *F. necrophorum* or another *Fusobacterium* sp. [13]. Some other microorganisms associated with Lemierre's syndrome include *Streptococcus*, *Staphylococcus*, *Eikenella corrodens*, *Porphyromonas asaccharolytica*, and *Bacteroides*. Isolation of microorganisms can be from blood or sites of metastatic infections. It is not uncommon to have negative or mixed growth on culture with this syndrome [13].

*P. asaccharolytica* is a rare etiological agent of Lemierre's syndrome. Prior to 1988, *Porphyromonas* was considered to be a subspecies of *Bacteroides melaninogenicus*. Classification of *Bacteroides* species was revised, and a new genus of *Porphyromonas* was proposed to include pigmented species without saccharolytic activity [6, 14]. *P. asaccharolytica* is a black-pigmented Gram-negative, obligate anaerobe that belongs to the family *Bacteriodaceae*. *P endodontalis* and *P gingivalis* are other members of the genus *Porphyromonas* present in humans [15]. *P. asaccharolytica* is more common in urogenital apparatus and gastrointestinal tract, while *P. endodontalis* and *P. gingivalis* are more commonly found in oral cavity [5]. To the best of our knowledge, there have been 3 other cases of Lemierre's syndrome reported in the literature in which *P. asaccharolytica* was the causative agent [5–7]. Lemierre syndrome being caused by an organism that is not usually found in oral cavity is an interesting finding.

The pathophysiology of this disease is not completely known. It has been postulated that there is a preceding infection by some other organisms, likely viral which impedes the local defense and facilitates invasion into pharyngeal space. Further spread can be through hematogenous, lymphatic, or direct through fascial plains between tonsils and parapharyngeal space [11]. Once the internal jugular vein is involved, metastatic infections to other sites such as lungs, bones, or brain can occur.

Pleuropulmonary complications can be present in more than 90% of cases with metastatic infections and can be in the form of septic lung emboli, lung abscess, pleural effusions, and/or empyema in various combinations [13]. Extrapulmonary manifestations include septic arthritis, muscle abscess, osteomyelitis, liver abscess, skin abscess, cerebral abscess, meningitis, and endocarditis. Renal failure and DIC can also be present in a small subset of patients [11].

The CT scan of the neck with contrast is the investigation of choice for diagnosis of this syndrome [16]. Doppler ultrasound is less sensitive and more dependent on the skill of operator. Findings on ultrasound include noncompressible low-level echos in the affected internal jugular vein associated with venous distention and absence of flow. It can also be considered for serial assessment of the clot after initial diagnosis [12].

Antibiotic therapy should be guided based on microbial isolation from blood or other sources. Surgical drainage should be considered in all parapharyngeal, cervical, or mediastinal abscesses. Lemierre's syndrome has traditionally been treated with penicillins, clindamycin, and/or metronidazole. *β-*lactamase-resistant strains have been found recently [11]. Metronidazole has good coverage for oropharyngeal anaerobes, but monotherapy is not recommended due to frequently present coinfections [17]. Patients are usually treated with a combination of beta-lactamase-resistant penicillins and metronidazole. Also, due to intraluminal spread of infection, Lemierre's syndrome is usually treated with a prolonged antibiotic course of 3 to 6 weeks, with intravenous antibiotics being preferred [3, 4]. Ligation of the internal jugular vein is historic and is rarely indicated in patients with persistent septic embolization despite antibiotics. Due to rarity of this disease, there have been no controlled studies to evaluate the role of anticoagulation in septic thrombophlebitis of the internal jugular vein. It is reserved for a specific subset of cases, in which benefits outweighs inherent risk of anticoagulation therapy. Patients with thrombus progression, as in our case, can be considered for anticoagulation therapy. Patients are usually anticoagulated with warfarin for 3 months and bridged with heparin [4]. Due to potential interaction of metronidazole with warfarin, INR needs to be monitored closely.

There was a mortality exceeding 90% in the preantibiotic era from a rapidly progressive disease course [3, 4]. In more recent reviews, mortality has been reported to be around 4–12%, which is still considerably high [10].
